# Luteolin 4′-Neohesperidoside Inhibits Clinically Isolated Resistant Bacteria In Vitro and In Vivo

**DOI:** 10.3390/molecules28062609

**Published:** 2023-03-13

**Authors:** Riham A. El-Shiekh, Mai A. Elhemely, Ibrahim A. Naguib, Sarah I. Bukhari, Rana Elshimy

**Affiliations:** 1 Department of Pharmacognosy, Faculty of Pharmacy, Cairo University, Kasr el Aini St., Cairo 11562, Egypt; 2 School of Medical Sciences, Faculty of Biology, Medicine & Health, The University of Manchester, Manchester M20 4GJ, UK; 3 Department of Pharmacology and Toxicology, Faculty of Pharmacy, Beni-Suef University, Beni-Suef 62514, Egypt; 4 Department of Pharmaceutical Chemistry, College of Pharmacy, Taif University, P.O. Box 11099, Taif 21944, Saudi Arabia; i.abdelaal@tu.edu.sa; 5 Department of Pharmaceutics, College of Pharmacy, King Saud University, Riyadh 11451, Saudi Arabia; sbukhari@ksu.edu.sa; 6 Department of Microbiology and Immunology, Faculty of Pharmacy, Ahram Canadian University, Giza 12451, Egypt; rana.elshimy@acu.edu.eg; 7 Department of Microbiology and Immunology, Egyptian Drug Authority, Giza 12511, Egypt

**Keywords:** STEC O111, *K. pneumoniae*, antimicrobial resistance, luteolin 4′-neohesperidoside, flavonoids, antibacterial

## Abstract

Multidrug resistance (MDR) pathogens are usually associated with higher morbidity and mortality rates. Flavonoids are good candidates for the development of new potential antimicrobials. This research investigated whether luteolin 4′-neohesperidoside (L4N) has antibacterial and synergistic activities against four antibiotic-resistant pathogens: methicillin-resistant *Staphylococcus aureus* (MRSA), *Klebsiella pneumoniae*, *fos*A-positive shiga toxin producing the *Escherichia coli* serogroup O111 (STEC O111), and *Bacillus cereus*. In vitro antimicrobial susceptibility testing revealed highly potent anti-MRSA (MIC of 106.66 ± 6.95 µg/mL), anti-*K. pneumoniae* (MIC of 53.33 ± 8.47 µg/mL) and anti-STEC O111 (MIC of 26.66 ± 5.23 µg/mL) activities. Significant synergistic combination was clearly noted in the case of gentamycin (GEN) against Gram-negative bacteria. In the case of *B. cereus*, the combination of vancomycin (VAN) with L4N could efficiently inhibit bacterial growth, despite the pathogen being VAN-resistant (MIC of 213.33 ± 7.9 µg/mL). In vivo evaluation of L4N showed significant decreases in *K. pneumoniae* and STEC shedding and colonization. Treatment could significantly diminish the levels of pro-inflammatory markers, tumor necrosis factor-alpha (TNF-α), and immunoglobulin (IgM). Additionally, the renal and pulmonary lesions were remarkably enhanced, with a significant decrease in the bacterial loads in the tissues. Finally, this study presents L4N as a potent substitute for traditional antibiotics with anti-STEC O111 and anti-*K. pneumoniae* potential, a finding which is reported here for the first time.

## 1. Introduction

In 2018, the World Health Organization (WHO) stated that the number of antibiotic-resistant pathogenic bacteria had drastically risen to reach a high-risk level that required global cooperation. More than 2.8 million people are infected with antibiotic-resistant bacteria every year in the United States, with about 35,000 dying as a result [1]. These superbugs are expanding globally, and the endless emergence of new resistance mechanisms challenges our ability to treat common infectious diseases and some nosocomial infections. The major trigger behind this crisis is the misuse of limited antibiotic sources in humans and animals. Likewise, the rate of new antibiotic development does not meet the growing rate of resistance.

Consequently, in addition to enhancing general awareness of proper antibiotic use, the search for novel antibiotics should be a priority for many research groups in the field of drug discovery [2]. It is worth noting that different classes of natural products, such as steroids, alkaloids, terpenoids, flavonoids, anthraquinone, tannins, saponins, etc., have been recommended to tackle the multidrug-resistant bacterial strains due to their potential pharmacological effects [3]. Most of the commonly used antibiotics are derived from microbial sources, while the plant-based ones have generally been utilized in recent years, during which several potential candidates have shown promising actions that can be further established and translated into clinical therapeutics [4].

Flavonoids are a large group of phytocompounds with different structures. As a main dietary component, flavonoids are reported to have health-promoting activities due to their high antioxidant capacity in both in vivo and in vitro models. They can be used in the management of degenerative diseases such as cardiovascular diseases, cancers, and other age-related diseases [5]. This is in addition to their protective effects against many infections (bacterial and viral diseases), such as those found in honey and propolis. They are reported to be largely responsible for this plant’s antimicrobial effects, with a strong background of use in clinical trials as antibiotics alone or in combination with conventional antibiotics [6,7].

Several studies have demonstrated the antibacterial effects of flavonoid-rich extracts and pure flavonoids. Luteolin (flavones) isolated from plants showed promising antibacterial activities against STEC O111, MRSA, *K. pneumoniae*, and *B. cereus* [8].

Luteolin-4′-*O*-neohesperidoside (L4N), i.e., luteolin-4′-*O*-[α-(L-rhamnopyranosyl-(1→2)-*β*-D-glucopyranoside)], was previously isolated from the genus *Caralluma*, showing significant anti-inflammatory and antinociceptive actions [9], and it also showed antibiofilm activity against an MRSA skin infection model [10]. Luteolin showed potent antimicrobial activity against *E. coli*, *K. pneumoniae*, MRSA, *Trueperella pyogenes*, and *Mycobacterium tuberculosis*. In addition, luteolin-7-*O*-β-D-glucuronide was reported to have potential activity against Gram-negative bacteria [11,12,13,14]. Additionally, flavones such as luteolin were found to strongly reduce *E. coli* macro-colony biofilms [8,15]. Thus, our study was designed to document the benefacial effects of flavones so as to combat resistant bacteria.

STEC is one of the pathogenic *E. coli* strains, among which certain strains have been found to be more virulent to humans, especially those belonging to serogroups O111 and O157. In this context, STEC O111 is a major public health concern in both developed and developing countries [3]. Enterohemorrhagic patients infected with STEC O111 are at a high risk of developing life-threatening extraintestinal complications, such as hemolytic uremic syndrome, hemorrhagic cystitis, and acute renal failure [4].

For uncomplicated cystitis caused by STEC, the main first-line empirical treatment is fosfomycin [16]. Fosfomycin is a broad-spectrum bactericidal antibiotic and among the most active agents used for sparing carbapenems in extended-spectrum b-lactamase (ESBL)-producing isolates and the treatment of carbapenem-resistant Enterobacteriaceae in combination with colistin [17]. The treatment of UTIs caused by STEC O111 is freuqnetly hindered by fosfomycin resistance, which emerges via the production of *fos*-A, a glutathione S-transferase that inactivates fosfomycin through the addition of a glutathione residue [18].

*K. pneumoniae* is a Gram-negative bacterium that causes pneumonia, intra-abdominal and blood infections, meningitis, pyogenic liver abscesses, and prominent nosocomial infections, with high mortality and morbidity rates in hospitalized and immunocompromised patients [5]. The expansion of multidrug-resistant (MDR) *K. pneumoniae* has resulted in an urgent need for the development of new, potent antimicrobial agents [6].

*B. cereus*, a volatile human pathogen, is an etiological negotiator of localized wounds; anthrax-like progressive pneumonia; intestinal, non-intestinal, and ocular infections; fulminant sepsis; and an overwhelming number of central nervous system infections, predominantly in immunosuppressed individuals and neonates [7,8].

Methicillin-resistant *Staphylococcus aureus* (MRSA) is a prominent agent of community- and health-care-associated infections (CAIs and HAIs) [9], ranging from superficial soft tissue infections (SSTI) to toxic shock syndrome (TSS), sepsis, and death [10].

**Figure 1:**
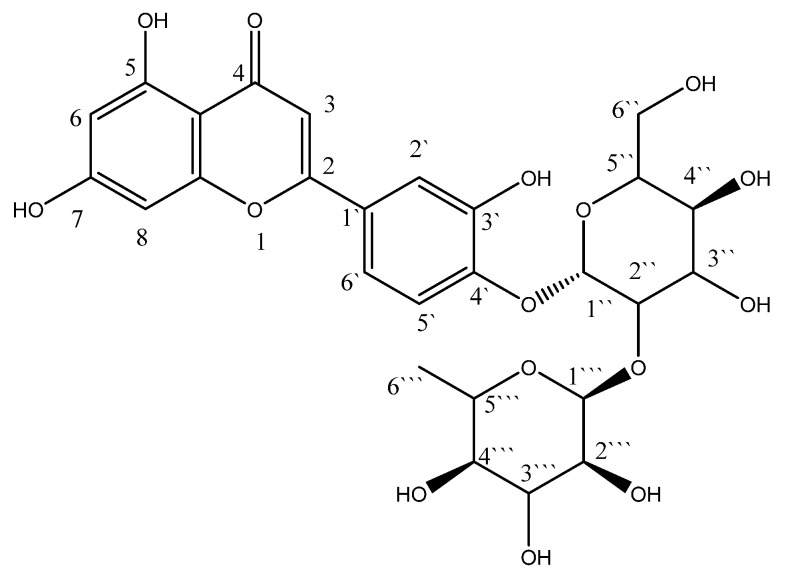
Structure of luteolin 4′-neohesperidoside (L4N).

The rise in resistance to various classes of antibiotics among the previously mentioned strains is a troublesome challenge faced by healthcare providers and hospital infection control units worldwide. The SARS-CoV-2 pandemic had crucial impacts on global health and economies [11]. In addition, it lent credence to the need to develop novel antimicrobials to be used solely or combined with traditional antibiotics. This study aimed to investigate the antimicrobial potential of luteolin 4′-neohesperidoside, L4N (Figure 1), against four MDR clinical isolates via in vitro and in vivo assessments.

## 2. Results

Flavonoids play an important role in decreasing the risk of several diseases associated with a diet rich in plant-derived foods. They are also common phytoconstituents used in traditional medicine for the treatment of a wide range of diseases. The emergence of multidrug resistance (MDR) and extensive drug resistance (XDR) among clinical isolates poses a significant challenge to conventional antibiotics, creating the antimicrobial resistance (AMR) crisis. In our study, we isolated a compound identified as luteolin 4′-neohesperidoside (L4N) and investigated its effects against resistant strains.

### 2.1. Spectral Data

#### Compound **L4N** (CD_3_OD)

^1^HNMR; 6.63 (s, H3), 6.23 (d, J = 2.2 Hz, H6), 6.47 (d, J = 2.2 Hz, H8), 7.49 (d, *J* = 2.2, 8.8 Hz, H2′), 7.46 (dd, *J* = 2.2, 8.8 Hz, H5′), 7.28 (d, J = 8.8 Hz, H6′), 5.21 (s, H1″), 5.29 (d, j = 1.8 Hz, H2″), 4.58 (s, H1‴), 3.17–4.02 (m, sugars).

^13^CNMR; 164.34 (C1&10), 103.86 (C2), 182.49 (C3), 158.47 (C4), 101.86 (C5), 162.77 (C6), 93.75 (C7), 137.47 (C8), 104.12 (C9), 125.86 (C1′), 116.39 (C2′), 148.31 (C3′), 147.76 (C4′), 113.61 (C5′), 118.72 (C5′), 101.74 (C1″), 79.57 (C2″), 77.21 (C3″), 70.82 (C4″), 76.76 (C5″), 60.82 (C6″), 99.48 (C1‴), 70.84 (C2‴), 72.58 (C3‴), 69.18 (C4‴), 69.78 (C5‴), 16.46 (C6‴) [19].

### 2.2. In Vitro Study

L4N showed inhibition zones of 20.6 ± 2.08 mm and 18.00 + 0 mm against STEC 0111 and *K. pneumoniae*, respectively, which are far higher than those of AK and GEN, being 0.0 ± 0.0 and 17.0 ± 1.00 mm, respectively. This indicates the promising antimicrobial activity of the compound (Table 1). There were highly significant differences between groups in the results for the inhibition zones of the antibiotics and L4N in regard to the MDR Gram-negative bacteria (MDR *K. pneumoniae* and STEC O111).

On the other hand, for the Gram-positive bacteria, L4N exhibited inhibition zones of 19.0 ± 0 mm and 6.0 ± 1.0 mm against MRSA and MDR *B. cereus*, respectively (Table 2). There were highly significant differences between groups in the results for the inhibition zones of the antibiotics and L4N in regard to the MDR Gram-positive bacteria (MDR *B. cereus* and MRSA) at a *p* value ≤ 0.05.

**Figure 2:**
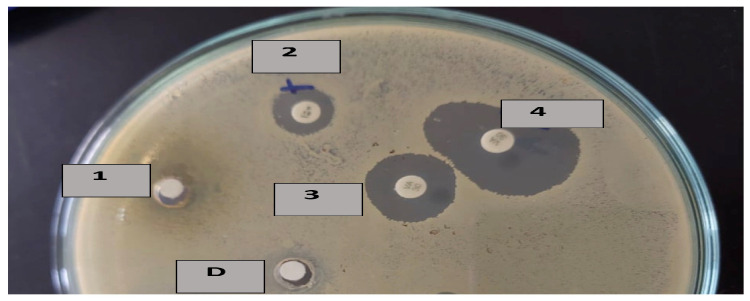
Inhibition zones of D: DMSO for 1: luteolin 4′-neohesperidoside (L4N) alone, 2: penicillin combined with L4N, 3: vancomycin alone, and 4: vancomycin combined with L4N.

A potent synergistic effect was noticed upon the combination of L4N with GEN against STEC and with VAN against MRSA and *B. cereus*, which was resistant to L4N, and for VAN used solely (Figure 2). These results agree with Amin et al. [20], who reported luteolin’s synergistic effect when combined with ceftriaxone and imipenem, ampicillin, oxacillin, and GEN against MRSA.

The MIC values of L4N against the tested Enterobacteriaceae were 26.66 µg/mL for STEC and 53.33 µg/mL for MDR *K. pneumoniae* (Table 3). 

The samples showed lower activity against the Gram-positive strains, with MIC values of 106.66 μg/mL and 213.33 μg/mL against MRSA and B. cereus, respectively (Table 4).

### 2.3. In Vivo Study

In this study, a model of *K. pneumoniae*-induced sepsis and germ-free mouse models were used to evaluate the antimicrobial activities of L4N against *K. pneumoniae* and STEC O111, respectively. When performing the in vivo assessment, both positive control groups died before the end of the experiment. Postmortem testing confirmed that all the mice were infected with high CFU loads, indicating that their death was caused by infection.

**Figure 3:**
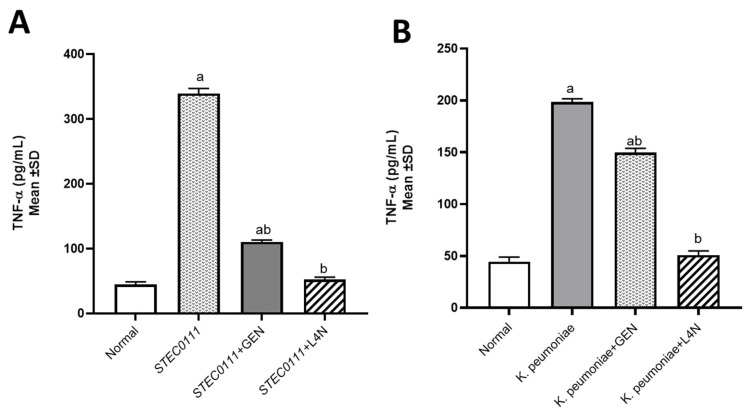
Tumor necrosis factor-alpha (TNF-α, pg/mL) levels in mice infected with MDR *K. pneumoniae* (**A**) and *E. coli* O111 (**B**) and then treated with luteolin 4′-neohesperidoside (L4N) and gentamycin (GEN). ^a^ Significant difference from normal control group at *p* < 0.05. ^b^ Significant difference from infected group at *p* < 0.05. ^ab^ Significant difference from normal control group and infected group at *p* < 0.05.

**Figure 4:**
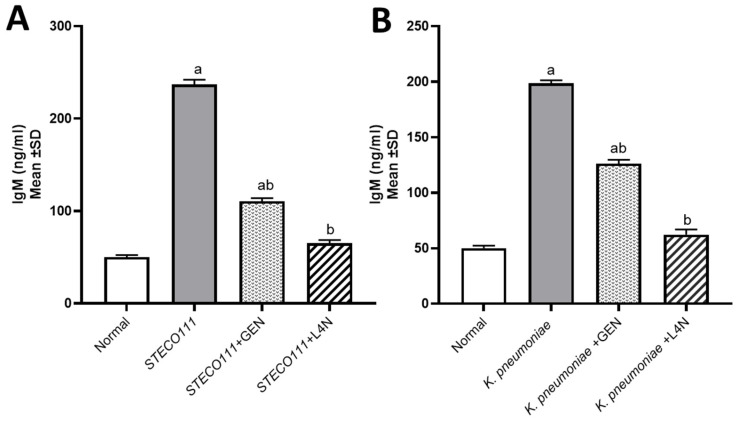
Immunoglobulin M (IgM, ng/mL) levels in the model of mice infected with MDR *K. pneumoniae* (**A**) and *E. coli* STEC O111 (**B**) and then treated with luteolin 4′-neohesperidoside (L4N) and gentamycin (GEN). ^a^ Significant difference from normal control group at *p* < 0.05. ^b^ Significant difference from infected group at *p* < 0.05. ^ab^ Significant difference from normal control group and infected group at *p* < 0.05.

The levels of tumor necrosis factor-alpha (TNF-α) and immunoglobulin M (IgM) were assessed to determine whether *E. coli* and *K. pneumoniae* strains elicited a proinflammatory environment and to conduct a comparison between the positive control groups, which were untreated, and the groups treated with either GEN or L4N. All the mice treated with L4N showed normal values of TNF-α and IgM (Figure 3 and Figure 4).

**Figure 5:**
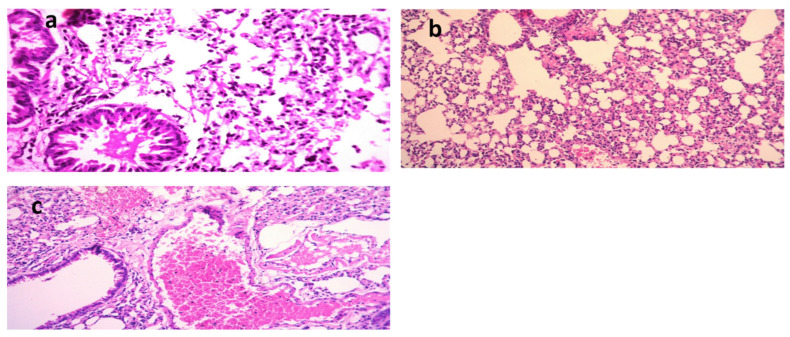
Histological sections of lungs stained with H&E and scoring of pulmonary lesions in a model of mice infected with MDR *K. pneumoniae*. (**a**) Mice infected with *K. pneumoniae* (X = 400), (**b**) mice infected with *K. pneumoniae* and then treated with gentamycin (GEN) (X = 200) [21], and (**c**) mice infected with *K. pneumoniae* and then treated with luteolin 4′-neohesperidoside (L4N) (X = 200).

The lungs of the *K. pneumoniae* (Figure 5a–c)-positive control mice showed dilated pulmonary blood vessels and focal hemorrhages in the interstitial tissue, as well as the alveolar lamina. Inflammatory infiltrate composed of histiocytes and polymorphonuclear cells was also detected. On the other hand, lungs of the mice infected with *K. pneumoniae* and then treated with Luteolin showed moderate infiltration of inflammatory cells, and emphysema of the pulmonary alveoli were observed. Lungs of the mice infected with *K. pneumoniae* and then treated with GEN showed emphysema of the pulmonary alveoli and mild inflammatory cell infiltration in the pulmonary tissues. In the group treated with luteolin, the scoring of the histopathological lesions (Table 5) revealed no signs of hemorrhage, no RBCs in the alveolar lamina, and no perivascular oedema.

After 24 h of *K. pneumoniae* inoculation, the mice showed signs of faster respiration, lower activity, disordered bristle or coats, and increased secretion around the eyes. These symptoms were enhanced after 48 h of inoculation, and the mice began to die. All the mice died within 168 h (7 days) in the model group, indicating that KNP inoculation induces a severe pulmonary inflammatory response, while in the L4N-treated group, pulmonary hemorrhage and the interalveolar thickness were remarkably reduced.

**Figure 6:**
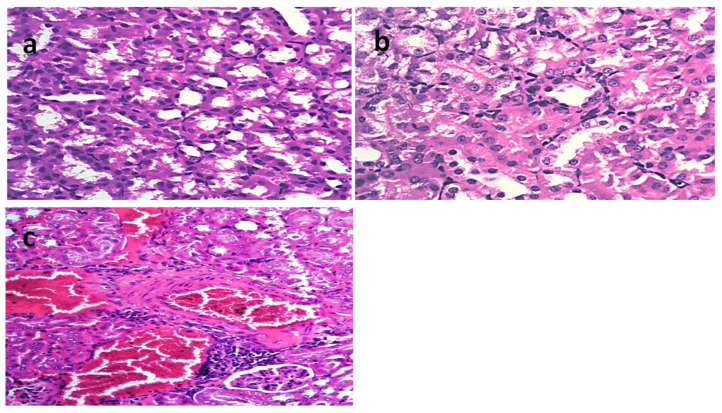
Histological sections of kidneys stained with H&E and scoring of renal lesions in a model of mice infected with *E. coli* O111. (**a**) Mice infected with *E. coli* O111 (X = 200), (**b**) mice infected with *E. coli* O111 and then treated with gentamycin (GEN) (X = 200), and (**c**) mice infected with *E. coli* O111 and then treated with luteolin 4′-neohesperidoside (L4N) (X = 200).

In the kidneys of the mice infected with STEC O111 (Figure 6a–c) (positive control group), the appearance of vacuoles of variable sizes in the cytoplasm of the tubular epithelium indicated necrotic changes. Moreover, characteristic histopathological changes, including coagulation necrosis with karyopyknotic nuclei, were also observed. However, the kidneys of mice infected with STEC and then treated with L4N showed interstitial inflammatory cell infiltration. Similarly, the kidneys of mice infected with STEC and then treated with GEN revealed focal inflammatory cell infiltration in the renal interstitial tissues and degenerative changes in the renal tubular epithelium. The scoring of histopathological lesions (Table 6) revealed that the mice treated with L4N showed no subcapsular hemorrhages, interstitial hemorrhages, necrobiotic changes in the tubular epithelium, interstitial inflammatory cell infiltration, or renal casts.

**Figure 7:**
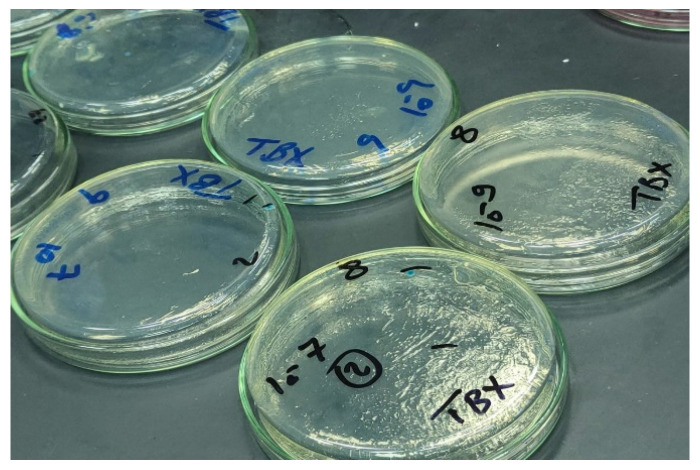
Quantification of STEC O111 on TBX media.

**Figure 8:**
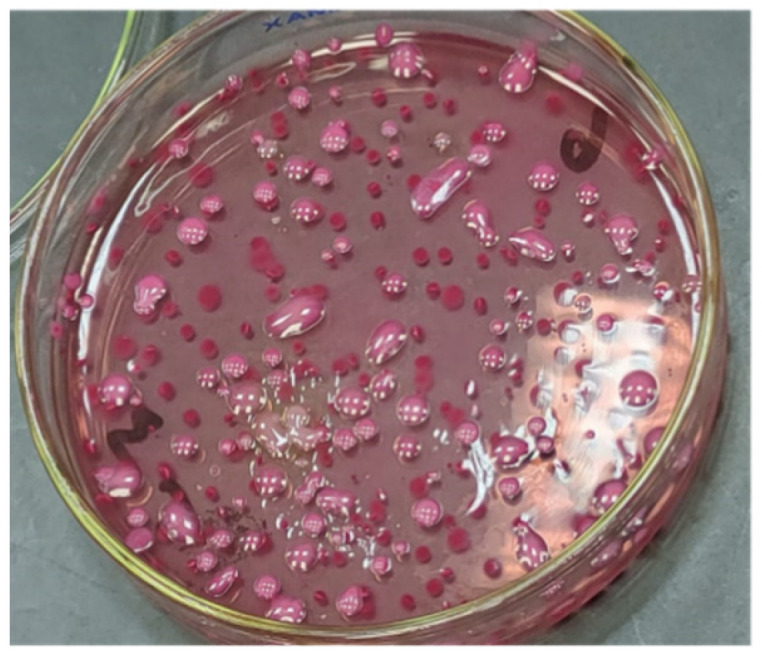
Quantification of *K. pneumoniae* on MacConkey agar media.

For the quantification of bacterial shedding, the bacteria in the feces were counted. The fecal count of STEC in the mice feces was reduced from 2 × 10^7^ to 3 × 10^2^ after 10 days of treatment with L4N, while that of the GEN-treated mice was reduced from 1 × 10^7^ to 4 × 10^3^ (Figure 7 and Figure 8). On the other hand, L4N and GEN could reduce the bacterial loads of *K. pneumoniae* from 19 × 10^6^ to 5 × 10^2^ and from 27 × 10^6^ to >250 × 10^2^, respectively (Table 7). The count of colonizing *K. pneumoniae* in the lungs and kidneys revealed a significant reduction in the bacterial count to 5 × 10^1^ in the lungs and to total eradication in the kidneys.

## 3. Discussion

The World Health Organization (WHO) has defined the fight against AMR as a priority and identified *K. pneumoniae* and *E. coli* as examples of critical, high-priority pathogens [22]. Although both are normal examples of human bacteria, they can easily develop AMR. Moreover, resistance to ampicillin, chloramphenicol, colistin, ciprofloxacin, fosfomycin, gentamicin, nitrofurantoin, tetracycline, trimethoprim/sulfamethoxazole, cefazolin, cefuroxime, and tobramycin has been rapidly increasing during the past few years [23].

Flavonoids, also known as polyphenolic compounds, have been widely tested for their antibacterial properties due to their ability to retard the growth of different pathogenic microorganisms, including MDR bacteria [3]. They are more active against Gram-negative bacteria than Gram-positive bacteria, reflecting a selective activity against Gram-negative bacteria. In particular, the Enterobacteriaceae showed the greatest sensitivity to flavonoids, for which the MIC was reported to be between 4 and 2048 μg/mL [24]. Our results presented herein collectively demonstrate that L4N effectively inhibits 0111 and *K. pneumoniae* infection in mice. The hydroxylation of C5, C7, C3′, and C4′ has been widely reported to increase the bacterial inhibition of flavonoids [3], and this finding is in agreement with our data. Furthermore, this is the first report on the role of L4N in the treatment of *E. coli* 0111 and MDR *K. pneumoniae*. The results obtained in the present study reveal the potent anti-*K. pneumoniae* and anti-STEC O111 effects of L4N. This observation is based on the results of a decreased bacterial burden and reduced level of the inflammatory mediator TNF-α and immunoglobulin (IgM).

## 4. Materials and Methods

### 4.1. Isolation of L4N from Phyllanthus Emblica

Dried fruits of *P. emblica* L. were obtained from a local market in Cairo, Egypt. The plants were kindly authenticated by Mrs. Therese Labib, Consultant of Plant Taxonomy at the Ministry of Agriculture and Former Director of El-Orman Botanical Garden. The dried plants (1 Kg) were ground and extracted using methyl alcohol by maceration for 3 days. To the concentrated alcoholic extract (150 g), 500 mL of water was added, and then the mixture was fractionated with dichloromethane (4 × 400 mL) and *n*-butyl alcohol (4 × 400 mL). A total of 20 grams of *n*-butyl alcohol extract was further subjected to polyamide chromatography (400 g of polyamide) by stepwise elution with aqueous methanol (0, 25, 50, 75 and 100%, *v*/*v*) to obtain five fractions. Fraction two (25% aqueous methanol) was evaporated to dryness under reduced pressure and yielded 750 mg. Then, the fraction was purified in a RP-18 silica gel column (1.5 × 25 cm) using MeOH: MeCN: H_2_O (7:3:0.5) to yield 130 mg of L4N, isolated as a yellow powder.

### 4.2. Characterization

NMR analysis was conducted on a Bruker High-Performance Avance III FTNMR spectrometer (^1^H-NMR: 400 MHz & ^13^C-NMR: 100 MHz) using TMS as the internal standard. Analytical TLC was performed on Merck TLC plates with KGF Silica gel 60 and KGF RP-18 Silica gel 60, Kenilworth, NJ, USA and the spots were visualized under UV light (254 & 365 nm) after spraying with aluminum chloride. Column chromatography was carried out on flash silica gel 60 (Merck, Kenilworth, NJ, USA, particle size 230, 400 mesh), RP-C18 (silica gel, 40 × 10^63^ mm; Merck, Kenilworth, NJ, USA), and polyamide (Merck, Kenilworth, NJ, USA).

### 4.3. Screening for Multidrug-Resistant Bacteria

On 5% blood agar plates, bacterial cultures were grown at 37 °C overnight, followed by inoculation on Mueller–Hinton agar. The susceptibilities of the clinical isolates were determined according to the CLSI protocol guidelines for *Enterobacteriaceae* [25]. The selected multidrug-resistant strains were screened for their resistance to more than two different classes of antibiotics following the disk diffusion method protocol (as in the CLSI guidelines) and WHO recommendations (CLSI, 2020; CLSI, 2014).

### 4.4. In Vitro Studies

The biological assay was carried out using previously phenotypically and genotypically identified bacterial strains, namely, multidrug-resistant STEC O111 and MRSA [23,26]. Multidrug-resistant *Bacillus cereus* and multidrug-resistant *K. pneumoniae* were isolated from the intensive care unit of a tertiary care center in Cairo [27].

Different culture media were used for each bacterium for identification and antibiotic susceptivity testing, as described previously [28]. TBX chromogenic agar media and MacConkey agar were used for the fecal counts of *E. coli* and *K. pneumoniae*, respectively [27].

The antimicrobial activities of L4N were first screened for their inhibitory zones by the agar disc diffusion method [26,29]. As a positive control for Gram-negative bacteria, gentamycin 10 µg (GEN) and amikacin 30 µg (AK) were used, while vancomycin (VAN) 30 µg and penicillin (PEN) 10 µg were used for the Gram-positive bacteria. On the other hand, a disc infused with 0.2% DMSO was used as the negative control. To test for synergistic or antagonistic effects between our tested compound and traditional antibiotics, they were combined with the compound. The assay was repeated using L4N alone, antibiotics alone, or a combination of both. The plates were incubated at 37 °C for 24 h, whereafter the zones of growth inhibition around the discs were observed and measured (mm). The tests were performed in triplicate and repeated twice [30,31,32] (CLSI, 2018, 2020).

The microbroth dilution method was implemented to determine the MIC [22]. Two-fold serial dilutions of the compound from 1024 µg/mL to 8 µg/mL (1024, 512, 256, 128, 64, 32, 16, and 8 μg/mL) were used. The MIC is the minimum bacteria growth inhibitory drug concentration. The positive controls (GEN or VAN) were serially diluted from 64 to 0.12 µg/mL. According to the results of the MIC, if the MIC was < 100 µg/mL, the compound was considered significantly active, and the compound was considered moderately active or weak if the MIC was ≥ 100 µg/mL [28].

### 4.5. Determination of the Safety Limit of L4N

The safety limit of L4N was determined by acute oral toxicity recording (LD50 value) conducted on six groups of 6- to 8-week-old CD-1 male mice weighing 30–40 gm (5 mice/group). The mice groups (1–6) received 100 µL of 8 µg/mL, 16 µg/mL, 32 µg/mL, 64 µg/mL, 128 µg/mL, and 256 µg/mL L4N orally once daily, respectively. The mortality of the mice was recorded after 48 h [33]. During the safety limit evaluation, no dead mice were observed throughout the experimental period, indicating that L4N is safe.

### 4.6. In Vivo Study

#### 4.6.1. STEC O111 Oral Infection Model

A previously described oral infection model of STEC O111 was implemented [34,35,36]. Briefly, 6- to 8-week-old CD-1 male mice weighing 30–40 gm were divided into four groups (10 mice/group), as follows: group 1, negative control (not infected or treated), group 2, positive control (infected and not treated), group 3 (infected and then treated with L4N), and group 4 (infected and then treated with GEN). All groups were fed orally by stomach gavage with an infection dose of 100 µL 10^8^ CFU of STEC O111 suspended in LB broth, except for the negative control group, which received 100 µL of LB broth with no bacterial cells, administered once daily for three successive days.

#### 4.6.2. *K. pneumoniae*-Sepsis-Induced Infection Model

The sepsis model was constructed as described previously, with minor modification [35]. CD-1 male mice aged 6 to 8 weeks old and weighing 30–40 gm (10 mice/group) were divided into four groups, as follows: group 1, negative control (not infected or treated), group 2, positive control (infected and not treated), group 3 (infected and then treated with L4N), and group 4 (infected and then treated with GEN). All groups were infected intraperitoneally with an infection dose of 100 µL 10^6^ CFU of *K. pneumoniae* suspended in LB broth, except for the negative control group, which received100 µL of LB broth with no bacterial cells, administered once daily for three successive days.

After three infection days, group 3, in the case of both the STEC and *K. pneumoniae* models, received 100 µL of 16 µg/mL and 32 µg/mL L4N (sub-MIC concentrations), respectively for ten days, administered orally once daily. Group 4 received 33 mg/kg of GEN I.P. once daily for three successive days. The control groups received 100 µL of saline. The animals were fed a commercial rodent diet with free access to drinking water. The mice were divided into seven groups, each comprising six mice.

Four mice from each group were dissected, and kidneys and large intestines were isolated from cages 1, 3, 6, and 7, while lungs were isolated from cages 1, 2, 4, and 5. All organs were subjected to further histopathological examination [21,37]. The organs were examined and compared to the control group. They were scored according to the extent of different pathological features.

#### 4.6.3. Biochemical Parameters

Serum samples stored at −80 °C were used for the measurement of the levels of tumor necrosis factor-alpha (TNF-α) and immunoglobulin M (IgM) using fluorescent-labelled microspheres (FluorMAP System; R&D Systems, Wiesbaden-Nordenstadt, Germany) and the Luminex 100 instrument (Luminex BV, Oosterhout, the Netherlands). All procedures closely followed the manufacturer’s instructions. 

### 4.7. Quantification of Bacterial Shedding

Bacterial shedding was calculated in CFU per gram of feces by plating on MacConkey agar media, as previously described [38].

This procedure followed the standard method of ISO 16649-2: 2001. The preparation of test samples, initial suspension, and dilution were performed according to ISO 6887 [39]. Two sterile Petri dishes were assigned for each dilution. Ten dilutions of feces from each group were prepared, and then 1 mL of each dilution was pipetted into the center of each dish. Afterwards, 15 mL of the TBX medium (44–47 °C) was poured into each dish and carefully mixed with the inoculum. The mixtures were allowed to solidify at room temperature. The period (or duration) between the end of the preparation of the initial suspension (or the 10^−1^ dilution, if the product was a liquid) and the moment when the medium was poured into the dishes did not exceed 15 min. Finally, the dishes were inverted and incubated at 44 °C for 18–24 h. The blue colonies of less than 150 CFU and less than 300 CFU in total (typical and non-typical) were counted.

The number (*N*) of *STEC 0001* per mL was calculated using the following equation:

*N* = *Σ^c^*/(*n*_1_ + 0.1 *n*_2_)*d*

Here, *Σ^c^* is the sum of the characteristic colonies counted on all the dishes retained, *n_1_* is the number of dishes retained in the first dilution, *n_2_* is the number of dishes retained in the last dilution, and *d* is the dilution factor corresponding to the first dilution.

### 4.8. Statistics

All quantitative results were analyzed using SPSS version 17.0 for Windows. Data are presented as the mean ± SD. Comparisons between multiple group means were performed using a one-way analysis of variance. Statistical significance was set as *p* ≤ 0.05.

## 5. Conclusions

It can be concluded that flavonoid-rich plants are good candidates for the treatment of MDR infections. This study presented luteolin 4′-neohesperidoside as a potent substitute for traditional antibiotics, as it showed anti-STEC O111 and anti-*K. pneumoniae* activities. This was confirmed by the observed reduction in the levels of pro-inflammatory markers and immunoglobulin, with a parallel improvement upon histopathological examination of renal and pulmonary lesions, which were remarkably enhanced, as well as a significant lowering of the bacterial loads in tissues.

## Figures and Tables

**Table 1:** Inhibition zones of antibiotics and luteolin 4′-neohesperidoside (L4N) in regard to MDR Gram-negative bacteria (STEC O111 and MDR *K. pneumoniae*).

Test Agent	Zone of Inhibition (Mean ± SD) (mm)	
	STEC O111	MDR *K. pneumonia*
DMSO	0.0 ± 0.0 ^a^ (R)	0.0 ± 0.0 ^a^ (R)
AK	0.0 ± 0.0 ^a^ (R)	0.0 ± 0.0 ^a^ (R)
GEN	17.0 ± 1.00 ^b^ (S)	8.66 ± 0.57 ^b^ (R)
L4N	20.6 ± 2.08 ^c^ (S)	18.00 ± 0.0 ^c^ (S)
AK + L4N	18.0 ± 0.0 ^b^ (S)	18.66 ± 1.15 ^c^ (S)
GEN + L4N	21.0 ± 1.00 ^c^ (S)	20.00 ± 0.0 ^d^ (I)

DMSO: dimethyl sulfoxide (negative control), AK: amikacin, GEN: gentamycin, R: resistant, S: sensitive, I: intermediate, L4N: luteolin 4′-neohesperidoside. There are significant differences between means with different letters at *p* ≤ 0.05.

**Table 2:** Inhibition zones of antibiotics and luteolin 4′-neohesperidoside (L4N) in regard to MDR Gram-positive bacteria (MRSA and MDR *B. cereus*).

Sample	Zone of Inhibition (Mean ± SD) mm	
	*B. cereus*	MRSA
DMSO	0.0 ± 0.0 ^a^ (R)	0.0 ± 0.0 ^a^ (R)
PEN	0.0 ± 0.0 ^a^ (R)	0.0 ± 0.0 ^a^ (R)
VAN	8.66 ± 0.57 ^b^ (R)	14.66 ± 0.57 ^b^ (S)
L4N	6.0 ± 1.0 ^c^ (R)	19.0 ± 0 ^c^ (S)
PEN + L4N	5.66 ± 0.57 ^c^ (R)	14.0 ± 0 ^b^ (I)
VAN + L4N	19.66 ± 1.52 ^d^ (S)	24.0 ± 1 ^d^ (S)

DMSO: dimethyl sulfoxide (negative contro), R: resistant, S: sensitive, I: intermediate, PEN: penicillin, L4N: luteolin 4′-neohesperidoside, VAN: vancomycin. There are significant differences between means with different letters at *p* ≤ 0.05.

**Table 3:** MIC of luteolin 4′-neohesperidoside (L4N) against MDR *E. coli* O 111 and MDR *K. pneumoniae*.

Sample	MIC (µg/mL)	
	STEC O111	MDR *K. pneumoniae*
NC	0.0 ± 0.0 ^a^	0.0 ± 0.0 ^a^
L4N	26.66 ± 5.23 ^c^	53.33 ± 8.47 ^c^
GEN	2.00 ± 0 ^b^	8.66 ± 1.15 ^b^

NC: negative control (plain media agar extracts in 0.2% DMSO), GEN: gentamycin, LN: luteolin 4′-neohesperidoside. There are significant differences between means with different letters at *p* ≤ 0.05.

**Table 4:** MIC of luteolin 4′-neohesperidoside (L4N) against MRSA and B. cereus.

Sample	MIC (μg/mL)	
	MRSA	MDR *B. cereus*
NC	0.0 ± 0.0 ^a^	0.0 ± 0.0 ^a^
L4N	106.66 ± 6.95 ^c^	213.33 ± 7.9 ^c^
VAN	2.00 ± 0 ^b^	4.0 ± 0 ^b^

NC: negative control (plain media agar extracts in 0.2% DMSO), VAN: vancomycin, LN: luteolin 4′-neohesperidoside. There are significant differences between means with different letters at *p* ≤ 0.05.

**Table 5:** Scoring of pulmonary lesions.

	a	b	c	d
**Hemorrhage**	0	3	0	0
**Thickened interalveolar septa**	0	3	1	1
**RBCs in alveolar lamina**	0	3	0	0
**Perivascular oedema**	0	4	0	0

a = negative control, b = positive control, c = treatment with luteolin 4′-neohesperidoside (L4N), d = treatment with gentamycin (GEN).

**Table 6:** Scoring of renal lesions.

	a	b	c	d
**Subcapsular hemorrhage**	0	2	0	0
**Interstitial hemorrhage**	0	3	0	0
**Necrobiotic changes in tubular epithelium**	0	3	1	1
**Interstitial inflammatory cell infiltration**	0	5	1	2
**Renal cast**	0	2	0	0

a = negative control, b = positive control, c = treatment with luteolin 4′-neohesperidoside (L4N), d = treatment with gentamycin (GEN).

**Table 7:** Quantification of bacterial loads of STEC O111 and *K. pneumoniae* shed in the feces and bacterial colonization in the kidneys and lungs.

	**STEC O111 (CFU/g Feces)**				***K. pneumoniae* (CFU/g Feces) **			
	**Negative Control**	**Positive Control STEC O111**	**STEC O111 then L4N**	**STEC O111 then GEN**	**Negative Control**	**Positive Control *K. pneumoniae***	***K. pneumoniae* then L4N**	***K. pneumoniae* then GEN**
Day zero	2 × 10^4^	3 × 10^6^	4 × 10^4^	4 × 10^6^	36 × 10^1^	22 × 10^2^	16 × 10^2^	36 × 10^2^
Day 4 (before challenge and after treatment with streptomycin)	2 × 10^1^	1 × 10^2^	0	1 × 10^1^	10 × 10^1^	1 × 10^2^	1 × 10^1^	1 × 10^1^
Day 7 (after challenge)	2 × 10^1^	4 × 10^9^	2 × 10^7^	1 × 10^7^	10 × 10^6^	18 × 10^7^	19 × 10^6^	27 × 10^6^
Day 10 (after treatment, 3 successive days of treatment with GEN I.P. once daily)	2 × 10^1^	Death	10 × 10^3^	4 × 10^3^	7 × 10^8^	Death	12 × 10^4^	>250 × 10^2^
Day 17 (after treatment, 7 successive days of treatment with LN I.P. once daily)	3 × 10^1^	NA	3 × 10^2^	NA	NA	NA	5 × 10^2^	NA
Kidney	0	NA	0	1 × 10^1^	NA			
Lung	NA				0	>300 × 10^3^	5 × 10^1^	6 × 10^2^

CFU: colony-forming unit, GEN: gentamycin, NA: not applicable, L4N: luteolin 4′-neohesperidoside.

## Data Availability

Not acceptable.

## Sample Availability

Samples of the compound (L4N) are available upon request from the authors.
