# Correction: El-Shiekh et al. Luteolin 4′-Neohesperidoside Inhibits Clinically Isolated Resistant Bacteria In Vitro and In Vivo. *Molecules* 2023, *28*, 2609

**DOI:** 10.3390/molecules30061283

**Published:** 2025-03-13

**Authors:** Riham A. El-Shiekh, Mai A. Elhemely, Ibrahim A. Naguib, Sarah I. Bukhari, Rana Elshimy

**Affiliations:** 1Department of Pharmacognosy, Faculty of Pharmacy, Cairo University, Kasr el Aini St., Cairo 11562, Egypt; 2School of Medical Sciences, Faculty of Biology, Medicine & Health, The University of Manchester, Manchester M20 4GJ, UK; 3Department of Pharmacology and Toxicology, Faculty of Pharmacy, Beni-Suef University, Beni-Suef 62514, Egypt; 4Department of Pharmaceutical Chemistry, College of Pharmacy, Taif University, P.O. Box 11099, Taif 21944, Saudi Arabia; i.abdelaal@tu.edu.sa; 5Department of Pharmaceutics, College of Pharmacy, King Saud University, Riyadh 11451, Saudi Arabia; sbukhari@ksu.edu.sa; 6Department of Microbiology and Immunology, Faculty of Pharmacy, Ahram Canadian University, Giza 12451, Egypt; rana.elshimy@acu.edu.eg; 7Department of Microbiology and Immunology, Egyptian Drug Authority, Giza 12511, Egypt

## Missing Citation

In the original publication [1], “Eloutify, Y.T.; El-Shiekh, R.A.; Ibrahim, K.M.; Elshimy, R.; Avula, B.; Katragunta, K.; Khan, I.; Meselhy, M.R. Bioassay-guided isolation of Antimicrobial Components and LC/QToF Profile of *Plumeria obtusa*: Potential for the treatment of Antimicrobial Resistance. *ACS Omega* **2023**, *8*, 6476–6491” was not cited. The citation has now been inserted in the legend of Figure 5 and the last paragraph of Section 4.6.2 and should read:

**Figure 5.** Histological sections of lungs stained with H&E and scoring of pulmonary lesions in a model of mice infected with MDR *K. pneumoniae*. (**a**) Mice infected with *K. pneumoniae* (X = 400), (**b**) mice infected with *K. pneumoniae* and then treated with gentamycin (GEN) (X = 200) [21], and (**c**) mice infected with *K. pneumoniae* and then treated with luteolin 4′-neohesperidoside (L4N) (X = 200).

## The Last Paragraph of Section 4.6.2

Four mice from each group were dissected, and kidneys and large intestines were isolated from cages 1, 3, 6, and 7, while lungs were isolated from cages 1, 2, 4, and 5. All organs were subjected to further histopathological examination [21,37]. The organs were examined and compared to the control group. They were scored according to the extent of different pathological features.

## References

With this correction, the order of some references has been adjusted accordingly.

The authors state that the scientific conclusions are unaffected. This correction was approved by the Academic Editor. The original publication has also been updated.
